# Plasma proteomics reveals early, broad release of chemokine, cytokine, TNF, and interferon mediators following trauma with delayed increases in a subset of chemokines and cytokines in patients that remain critically ill

**DOI:** 10.3389/fimmu.2022.1038086

**Published:** 2022-11-30

**Authors:** Jillian Bonaroti, Isabel Billiar, Hamed Moheimani, Junru Wu, Rami Namas, Shimena Li, Upendra K. Kar, Yoram Vodovotz, Matthew D. Neal, Jason L. Sperry, Timothy R. Billiar

**Affiliations:** ^1^ Department of Surgery, University of Pittsburgh, Pittsburgh, PA, United States; ^2^ Pittsburgh Trauma and Transfusion Medicine Research Center, Division of Trauma and Acute Care Surgery, University of Pittsburgh, Pittsburgh, PA, United States; ^3^ Xiangya School of Medicine, Central South University, Changsha, China

**Keywords:** chemokine, proteomics, immune mediators, cytokine, traumatic injury, TNF, interferon

## Abstract

Severe injury is known to cause a systemic cytokine storm that is associated with adverse outcomes. However, a comprehensive assessment of the time-dependent changes in circulating levels of a broad spectrum of protein immune mediators and soluble immune mediator receptors in severely injured trauma patients remains uncharacterized. To address this knowledge gap, we defined the temporal and outcome-based patterns of 184 known immune mediators and soluble cytokine receptors in the circulation of severely injured patients. Proteomics (aptamer-based assay, SomaLogic, Inc) was performed on plasma samples drawn at 0, 24, and 72 hours (h) from time of admission from 150 trauma patients, a representative subset from the Prehospital Plasma during Air Medical Transport in Trauma Patients at Risk for Hemorrhagic Shock (PAMPer) trial. Patients were categorized into outcome groups including Early Non-Survivors (died within 72 h; ENS; n=38), Non-Resolvers (died after 72 h or required ≥7 days of intensive care; NR; n=78), and Resolvers (survivors that required < 7 days of intensive care; R; n=34), with low Injury Severity Score (ISS) patients from the Tranexamic Acid During Prehospital Transport in Patients at Risk for Hemorrhage After Injury (STAAMP) trial as controls. The major findings include an extensive release of immune mediators and cytokine receptors at time 0h that is more pronounced in ENS and NR patients. There was a selective subset of mediators elevated at 24 and 72 h to a greater degree in NR patients, including multiple cytokines and chemokines not previously described in trauma patients. These findings were validated in a quantitative fashion using mesoscale discovery immunoassays (MSD) from an external validation cohort (VC) of samples from 58 trauma patients matched for R and NR status. This comprehensive longitudinal description of immune mediator patterns associated with trauma outcomes provides a new level of characterization of the immune response that follows severe injury.

## Introduction

Trauma is a leading cause of morbidity and mortality worldwide, accounting for over 5 million deaths and costing an estimated $400 billion annually in medical costs and lost wages (1, 2). Immune dysfunction contributes to this morbidity and is associated with an excessive systemic inflammatory response and a sustained impairment in immune defenses (3–6). These responses are manifested as early organ injury and a sustained susceptibility to infection that goes beyond the organ dysfunction phase. The measurement of levels of circulating immune mediators has been used to assess the state of the immune system in injured humans (7–12).

In a recent review, we summarized the major findings regarding the association of cytokines and chemokines in the various outcomes experienced by trauma patients (7). Consistent findings include the early elevations in pleiotropic mediators such as IL-6 and IL-10 as well as the prototypic chemokines CXCL8 (IL-8), CXCL9 (MIG) and CCL2 (MCP1) (8, 12–15). These mediators have been shown to correlate with injury severity and adverse outcomes. Early elevations in mediators associated with tissue protection and repair, including IL-9, IL-17E (IL-25), IL-21, IL-22, IL-23, and IL-33 associate with an improved clinical course (16, 17). A third group of mediators typically associated with lymphocyte responses, including, IL-2, IL-4, IL-5, and IL-7 are also elevated later following injury and associate with improved outcomes (16). In contrast, it has been shown that IL-17A levels can associate with either worse or better outcomes, and its association with other mediators, such as IL-10 or GM-CSF, may be a key determinant of the roles of IL-17A after injury (17). While these studies reveal interesting patterns in mediator levels, a comprehensive assessment of immune mediator levels has been limited by the available assays. For example, only a partial subset of the known interleukins (n=40), chemokines (n=47), interferons (n=21), and TNF superfamily (TNFSF) members (n=19) have been measured in humans after trauma (18–24).

With advances in high-dimensional proteomic analysis, it is now feasible to measure entire families of proteins simultaneously (25). Here, we used longitudinal proteomic databases derived from the SomaLogic 7.5K Proteomic Platform (26) assay of plasma samples obtained from the Prehospital Plasma during Air Medical Transport in Trauma Patients at Risk for Hemorrhagic Shock(PAMPer) (27) and Tranexamic Acid During Prehospital Transport in Patients at Risk for Hemorrhage After Injury (STAAMP) (28) trials to characterize the protein immune mediator landscape in severely injured humans. This allowed the association of patterns of 115 immune mediators and 69 soluble cytokine receptors with patient outcomes. Both expected and novel patterns were observed, providing a more complete picture of mediator dynamics and insights into the nature of the evolving immune response after injury in humans.

## Materials and methods

### Patient enrollment and data collection

The PAMPer and STAAMP trials were approved by the IRB of University of Pittsburgh. Detailed information and the study protocol for the PAMPer Trial are available on https://clinicaltrials.gov/ct2/show/NCT01818427 and for the STAAMP trial on https://clinicaltrials.gov/ct2/show/NCT02086500. The PAMPer Trial received approval for Emergency Exception from Informed Consent (EFIC) from the Human Research Protection Office of the US Army Medical Research and Material Command. The analysis performed here is a secondary analysis of a published dataset using de-identified data and therefore, is not considered human subject research.

Analysis was performed on plasma samples collected on admission (0 h), 24 h, and 72 h post-admission from a subset of the PAMPer trial patient population. Only one sample (0 h) was analyzed for patients who died within 72 h of admission. The criteria for enrollment as well as the patient characteristics have been previously described (27). Trauma patients who had experienced at least one pre-hospital episode of hypotension (systolic blood pressure (SBP)<90 mmHg and heart rate (HR)>108/min, or SBP<70mmHg) were randomized to receive standard of care fluid resuscitation (crystalloid with or without packed blood cells) or 2 units thawed plasma (TP) during air-medical transport followed by standard of care of fluid resuscitation. Traumatic brain injury (TBI) was diagnosed at each enrollment site from the initial head CT scan.

For the current study, proteomic measurements were conducted on a representative cohort of 150 patients selected from the 194 PAMPer patients with available plasma samples. The 150 patient samples were chosen as a representative subset of patients that also had metabolome, lipidome, cytokine, and endotheliopathy marker data previously acquired. The multi-omic analysis of these patients has been previously published (26, 29), and the present study represents an analysis of four families of protein mediators present within the proteomic analysis. Twenty-nine low or no-injury trauma patients transported by air medical transport in the STAAMP trial (28), sampled upon arrival, were included as minimally injured (Injury Severity Score [ISS]=1 or less) controls. These control patients were enrolled in STAAMP based on broad inclusion criteria including SBP <90 *or* HR >110, but were ultimately uninjured. They were transported, evaluated in the trauma center, and underwent blood sample collection, thus exposing them to identical hospital based experiences as the injured PAMPer patients. Therefore this low or no-injury STAAMP cohort is a representative control population. The demographics of the STAAMP and PAMPer study populations are shown in **Supplemental Table 1**. The groups differ in age (36.6 vs 47.7 p=0.006) which must be considered when interpreting the data. Based on the survival status and length of stay in the intensive care unit (ICU), the total PAMPer cohort was further segregated into either Early Non-Survivors (ENS; patients who died within 72 h of admission), Resolvers (R, alive at 30 days and required <7 days in ICU), and Non-Resolvers (NR, died after 3 days or required ≥ 7 days in ICU) (see **Table 1**).

**Table 1:** Demographic data and clinical outcomes of patient groups.

	Early NS (n=38)	Resolving (n=34)	Non-Resolving (n=78)	p-value (NR v R)
DEMOGRAPHICS				
Age, years	49.4 ± 23.1	46.4 ± 18.4	46.4 ± 20.1	1
Sex, male/female	M=29, F=9	M=25, F=9	M=59, F=19	0.8124
Injury severity score	32.6 ± 19.0	19.9 ± 8.31	31.0 ± 13.6	<0.0001
Intervention arm	TP=12 ,C=26	TP=13 ,C=21	TP=40 ,C=38	0.11
Transport time (min)	44.1 ± 15.3	44.9 ± 19.0	47.5 ± 22.6	0.55
CLINICAL OUTCOMES				
Hospital LOS, days	1.24 ± 0.634	12.4 ± 8.91	21.4 ± 13.4	0.0005
ICU LOS, days	0.895 ± 0.798	3.35 ± 1.81	14.8 ± 8.72	<0.0001
Mechanical ventilation, days	1.18 ± 0.652	1.76 ± 1.56	11.1 ± 6.18	<0.0001

For the validation cohort (VC), we used a prospectively maintained clinical database and biobank of 58 polytrauma patients selected from 89 patients admitted through the Emergency Department (ED) at UPMC Presbyterian Hospital (Level 1 trauma center). To derive stringently matched sub-cohorts, 33 VC Resolvers were matched to 26 PAMPer Resolvers and 25 VC Non-Resolvers were matched to 39 PAMPer Non-Resolvers using IBM SPSS Statistics^®^ case-control matching (controlling for age, gender ratio, and injury severity based on ISS) (see **Table 2** and **Supplemental Table 4**).

**Table 2:** Demographic data and clinical outcomes of patient groups after matching for ISS.

	Resolving (n=26)	Non-Resolving (n=39)	p-value (NR v R)
DEMOGRAPHICS			
Age, years	47.0 ± 17.8	47.1 ± 16.3	0.9817
Sex, male/female	M=19, F=7	M=31, F=8	0.5479
Injury severity score	23.1 ± 6.03	23.6 ± 7.82	0.7716
Intervention arm	TP=13 ,SOC=13	TP=17 ,SOC=22	0.61
CLINICAL OUTCOMES			
Hospital LOS, days	13.0 ± 9.61	22.6 ± 15.1	0.0035
ICU LOS, days	3.65 ± 1.74	15.8 ± 9.35	<0.0001
Mechanical ventilation, days	2.04 ± 1.64	11.7 ± 6.64	<0.0001

### Study design and procedures

Upon hospital admission, clinical and demographic data were obtained from eligible patients: demographics—age and sex; clinical information—mechanism of injury, elapsed time from injury to the emergency room arrival, ISS, and head abbreviated injury scale (AIS); neurological status—level of consciousness categorized by the Glasgow coma scale (GCS), pupil size and reactivity, seizures, and alcohol level; clinical status—blood pressure, tracheal intubation, spontaneous vs. mechanical ventilation, oxygen saturation, and temperature; and medical history—past medical history, present medications including β-blockers and anticoagulants. Routine laboratory exams and imaging were also completed upon admission, including chest radiography and computerized tomography (CT) scans. All significant clinical events during the first 24 h were recorded, including, but not limited to, sepsis/infection, organ failure, any medical treatments administered, surgical procedures, and any other significant changes in clinical parameters. Patients were followed up to 28 days post-admission. Hospital and ICU lengths of stay as well as the cause of death (in those patients that died) were registered and classified.

### Blood sample collection

Venous blood samples were drawn as soon as possible after presentation to the trauma bay or emergency department (t = 0h), and again at 6h, 12h, 24h, and 72h post-injury. Samples were drawn into either 10 mL K_2_EDTA [with 4 mM sodium metabisulfite (Na_2_S_2_O_5_)] or 10-mL sodium heparin vacutainers (Vacutainer, Becton Dickinson, Rutherford, NJ). Specimens were immediately centrifuged at 1600×g for 15 min at 4°C; the plasma supernatant was then separated into six (1–2 mL) aliquots and frozen at −70°C until subsequent analysis.

### Global plasma proteomics assay

The proteomic profiles of the plasma samples were characterized using the SomaScan Assay (SomaLogic, Inc.; Boulder, CO, USA) capable of measuring levels of 7,596 human proteins in plasma. The assay is based on protein-capture reagents constructed by chemically modified, single-stranded DNA and uses chemically modified nucleotides to transform a protein signal into a nucleotide signal that can be quantified using relative fluorescence on microarrays (30). A total of 7,211 proteins (94.9%) passed the accepted range for the Hybridization and Calibration Scale factors (0.4-2.5 and 0.8-1.2, respectively). As noted previously, groups of samples were measured independently (29).

In order to perform a focused analysis of immune mediator proteins, 184 of the 7211 proteins measured and annotated as known immune mediators from families of interleukins (IL, n=40), C−C motif chemokine ligands (CCL, n=23), C-X-C motif chemokine ligands (CXCL, n=10), interferons (IFN, n=27), tumor necrosis factor superfamily members (TNF, n=15) and soluble receptors for cytokines (n=38), interferons (n=5), and TNF superfamily members (n=26) were extracted from the data set and analyzed. A complete list of the mediators and receptors included in the analysis is shown in **Supplemental Table 2**. For each analysis, all proteins with more than 20% of values missing were removed. To account for the possibility of a detection limit, the minimum value was used to infer the remaining missing values. Raw fluorescent intensity data were normalized by multiple hybridization and calibration scale factors for each sample.

### Targeted measurement of immune mediators

Quantitative measurement of immune mediators from the PAMPer and VC datasets have been published previously (13, 16, 31, 32). Measurements were performed using kits from the Luminex™ MAGPIX assay system (Austin, TX, Luminex™, Inc.) per the manufacturer’s instructions.

### Multiplex cytokine and chemokine measurements

Immunoreactive plasma levels of selected cytokines and chemokines were analyzed with Meso Scale Discovery (MSD, Meso Scale Diagnostics, Rockville, Md) 96-Well MULTI-SPOT^®^ Ultra-Sensitive Human Immunoassay Kits, using electrochemiluminescence detection on an MSD Sector Imager™ 6000 with Discovery Workbench software (version 3.0.18) (MSD^®^, Gaitherburg, MD, USA). Cytokines and chemokines were measured using the U-PLEX/R-PLEX Custom Biomarker (hu) assays. Analyte concentrations were calculated per the manufacturer’s protocol (MSD DISCOVERY WORKBENCH analysis software) and were considered detectable if both runs of each sample had a signal greater than the analyte- and plate-specific lower limit of detection (LLOD) (i.e., 2.5 standard deviations of the plate-specific blank). Analyte concentrations (pg/mL) from both runs of each analyte were averaged for analysis.

### Statistical analysis

To approximate a normal distribution, log2-transformed values of the normalized mediator measures were used for all downstream analyses. Either Student t-test or Mann-Whitney U test was used for normal and non-normal distributed data, respectively. Two-tailed *p*-values were calculated to assess the statistically significant difference between resolving versus non-resolving patient groups and injury severity-based groups. Pearson’s χ2 test was used for categorical variables. For near-normally distributed data, multiple group comparisons were conducted by the ANOVA test followed by the Tukey *post hoc* test. Non-normal distributions were assessed using the Kruskal-Wallis test followed by Dunn’s *post-hoc* test. In all analyses, the *p*-values were adjusted to account for multiple testing using the Benjamini-Hochberg false discovery rate correction.

### Visualizations

Standardized values (z-scores) across each category were used to generate heatmaps visualizing changes in mediator levels over time, based on either outcome or injury severity groups. All on-the-map comparisons between R and NR at each time point were performed using the Mann-Whitney U test.

To create volcano plots at each time point, the difference between the log2-transformed level of each mediator in NR and R patients was plotted on the x-axis. The y-axis plotted the log10-transformed value of adjusted *p*-values from the Mann-Whitney U test of between-group differences.

For mediators measured with the MSD assay, box and whiskers plots were created demonstrating median and IQR values of log2-transformed mediator levels at 0h, 24h, and 72h time points. Pairwise between-group differences in each chart were performed using Dunn’s *post hoc* method.

### Predictive modeling

A multistep machine learning pipeline was designed to assess the performance of relative immune mediator levels at the 72h time point in predicting eventual patient outcomes (resolving versus non-resolving status). We implemented an iterative sampling procedure to assure the model captured the underlying information and was not biased toward the PAMPer population’s hyper-specific features (i.e. noise).

For each iteration of the modeling procedures, we first obtained a random sample of 112 patients from the total population of 34 resolvers and 78 non-resolvers. As sampling was performed with replacement, it resulted in a bootstrap sample that had more than one instance of a random subset of individuals but did not include some others (i.e. *out-of-bag* individuals). Then, all 115 SomaLogic measurements for mediator values of sample instances were entered into a Least Absolute Shrinkage and Selection operator (LASSO) model. The selected mediators (variables passing LASSO with weights > 0) were subsequently used to train a Support Vector Machines model (with the linear kernel) that predicted the binary outcome (resolver vs. non-resolver) in the training sample. Finally, the performance of the trained model was tested on the out-of-bag individuals. The lack of signal leakage between the training and testing populations ensured the results were relatively robust to noise.

The process detailed above was performed 1000 times, providing 1000 Area Under the Receiver Operating Characteristics curve (AUROC) calculations that could be summarized as the median and 95% confidence interval for model performance evaluation. The significance test was performed on the difference between the mean AuROC value of the predictive model and a permuted control model whose training and evaluation followed the exact same steps; however, the outcome status of its training sample instances was randomly permuted for each iteration.

We computed a generic predictor importance score to further assess the importance of contributions from each mediator to the model’s predictions. For each predictor, the score was calculated by taking the quadratic mean of its weights in all iterations of the SVM model. The highest score was set as 100 and all other scores were proportionally scaled.

To assess how the addition of demographic and clinical variables could affect the model’s predictive capability and feature importance metrics, all steps were replicated to develop and present a secondary model that used ISS, age, and gender in addition to the mediator values for outcome prediction.

Using both sets of variables, Partial Least Squares Discriminant Analysis (PLS-DA) was implemented to visualize the discriminative ability of both the mediator-only and secondary prediction models.

All statistical analyses and visualizations were performed using pandas, numpy, matplotlib, seaborn, scipy, sklearn, and statsmodels packages of Python programming language version 3.9.

## Results

### Patient demographics and clinical characteristics of the unmatched cohort

We first performed an analysis of circulating immune mediators (n=115) and soluble immune mediator receptors (n=69) on plasma samples from representative cohorts of patients from the PAMPer trial. Patients were segregated into 3 groups: Resolving (R, n=34), Non-resolving (NR, n=78), and Early Non-survivors (ENS, n=38) as described in the Methods. The demographics and clinical characteristics of the 3 groups are shown in **Table 1**. There were no significant differences in age or sex between groups; however, NR patients had higher Injury Severity Scores (ISS) than R patients (31.0 +/- 13.6 vs 19.9 +/- 8.31, *p=*0.0001). NR patients also had longer hospital LOS, ICU LOS, and days of mechanical ventilation. The proportion of patients receiving standard of care versus thawed plasma intervention and transport time were not significantly different between groups. Minimally injured patients from the STAAMP trial with low ISS (Mean (range)=0.5 (0–1) were used as a comparative control.

### The extensive release of immune mediators and cytokine receptors at time 0 h is more pronounced in NR and ENS, with subsets of mediators elevated at 24 and 72 h

**Figure 1:**
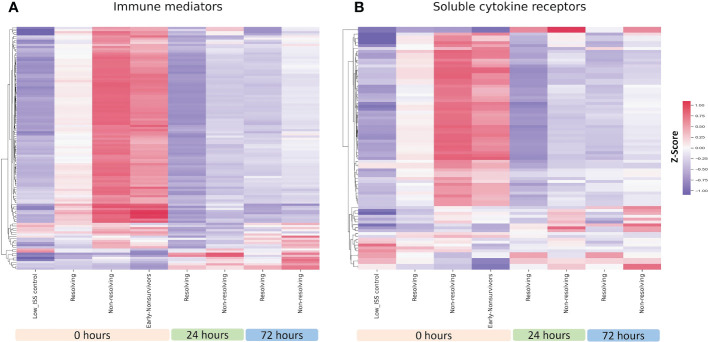
Heatmap illustrating normalized z-scores for all 115 assayed immune mediators **(A)** and 69 soluble cytokine receptors **(B)** in ENS (n=38), NR (n=78), and R (n=34) patients at 0, 24 and 72 h with low ISS (ISS of 1 or less, n=29) patients as controls.

We used heatmaps of normalized z-scores of immune mediator and receptor levels to visualize the patterns of mediators and soluble receptors in circulation. Shown in **Figure 1A** is a heatmap depicting the relative levels of 115 immune mediators for R, NR, and ENS patients at 0, 24, and 72 h. At 0 h, the time of admission, most of the assayed immune mediators were higher than the levels in low ISS control patients across all three groups. Notably, these levels were dramatically higher in NR and ENS patients in comparison to R patients. This suggests a pattern of early, massive release of immune mediators following trauma that is more pronounced in patients that die early or that resolve from critical illness slowly. There was a small subset of mediators that were higher in the controls at time 0 h and included 3 chemokines and 2 cytokines out of the 115 mediators. A subset of immune mediators that were low at time 0 h increased at 24 and 72 h in both R and NR but was more pronounced in NR patients. We also assessed the relative levels of 69 soluble immune mediator receptors and observed a similar pattern with most receptors high at time 0 h in the NR and ENS groups in comparison to R patients. Similar to the mediator pattern, a subset of receptors that were low at time 0 h in all groups increased at 24 and 72 h in both R and NR but were more pronounced in NR patients (**Figure 1B**).

### Patient demographics and clinical characteristics of an ISS-matched cohort

**Figure 2:**
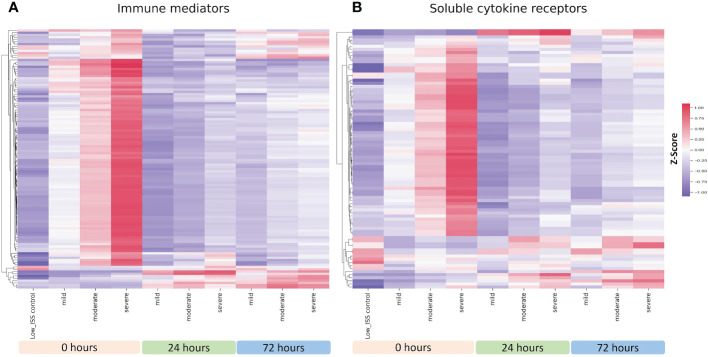
Heatmap illustrating normalized z-scores for all 115 assayed immune mediators **(A)** and 69 soluble cytokine receptors **(B)** in patients with mild (n=23), moderate (n=49), and severe injury (n=78) at 0, 24 and 72 h with low ISS (ISS of 1 or less, n=29) patients as controls.

With the pattern differences demonstrated between R and NR patients in our initial analysis, we questioned if these findings are a function of differing ISS between R and NR groups. As shown in **Figure 2**, dividing patients into mild (1-15), moderate (16-24), and severe (≥25) ISS groups revealed an injury severity-based change in mediator and cytokine receptors levels in the circulation, with the highest levels observed in the high ISS group at 0 h. We therefore carried out propensity matching to obtain groups of NR and R patients matched for ISS, age, and sex. The demographics and clinical characteristics of the two groups are shown in **Table 2**. There were no significant differences in age, sex, or ISS between groups, with mean ISS 23.1± 6.03 for R (n=26) versus 23.6 ± 7.82 for NR (n=39). The proportion of patients receiving standard of care (SOC) or thawed plasma (TP) intervention was not significantly different between groups. NR patients had longer hospital LOS, ICU, LOS, and days of mechanical ventilation.

### Immune mediator and cytokine receptor patterns are replicated in an ISS-matched cohort

**Figure 3:**
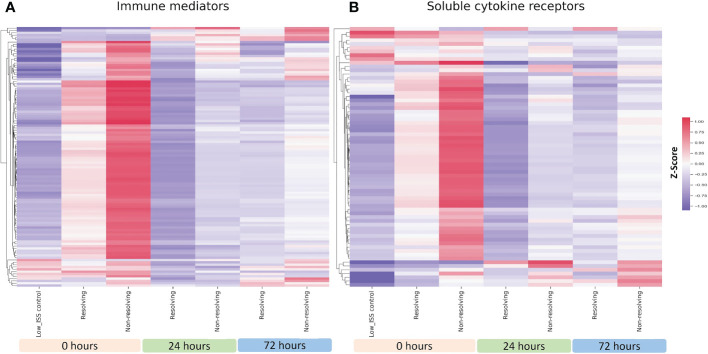
Heatmap illustrating normalized z-scores for all 115 assayed immune mediators **(A)** and 69 soluble cytokines **(B)** in ISS matched NR (n=39) and R (n=26) patients at 0, 24 and 72 h with low ISS (ISS of 1 or less,.

Shown in **Figure 3A** is a heatmap including all the measured immune mediators for R and NR patients at 0, 24, and 72 h matched for ISS, age, and sex. Similar to the unmatched analysis, at 0 h in both groups, the majority of the assayed immune mediators were high in comparison to low ISS controls, but levels were notably higher in NR patients. At 24 and 72 h, a subset of immune mediators that were low at time 0 h increased in both R and NR but was more pronounced in NR patients. Shown in **Figure 3B** is a heatmap including soluble cytokine receptors for the ISS matched analysis. Again, mirroring the unmatched analysis, most receptors were higher at time 0 h in the NR and ENS groups in comparison to low ISS controls and a subset of receptors that were low at time 0h in all groups increased at 24 and 72h in both R and NR but were more elevated in NR patients. The patterns of the ISS matched cohort demonstrated in **Figure 3** are consistent with the unmatched analysis, suggesting that these patterns are independent of ISS, age, or sex.

### Chemokines, Interleukins, and TNF superfamily members have significant fold-change differences between R and NR patients

**Figure 4:**
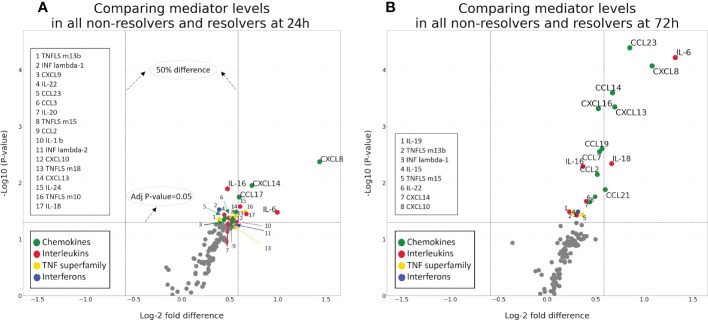
Volcano plots demonstrating the log-2fold change and significance of difference between NR (n=78) and R (n=34) patients at 24 **(A)** and 72 **(B)** h.

To better define the differences in immune mediator patterns between groups, we performed significance testing on the fold-change differences between NR and R patients at 24 and 72 h. **Figure 4** shows volcano plots demonstrating the log-2 fold change and the log10 *p*-value for mediators at 24 h (A) and 72 h (B). The upper right quadrant of each plot includes mediators with a fold change of at least 1.5 with a *p*-value <0.05. This analysis reveals several mediators in the chemokine and interleukin families with a significant fold increase when comparing NR to R patients. Among those with the greatest fold change and *p*-value <0.05 at 72 h are IL-6, CXCL8, CCL23 (also known as macrophage inflammatory protein 3, MIP-3) CXCL13, CCL14, CCL21, and IL-18. At 24h, those with the greatest fold change and *p-*value <0.05 are CXCL8, CXCL14, CCL17, IL-6, IL-18, and IL-24 (**Supplemental Table 3**). Similar patterns are observed when this analysis is performed on the ISS, age, and sex matched cohort (**Supplemental Figure 1**). In both analyses, chemokines dominate the mediators with significant fold- increases when comparing NR to R. These findings demonstrate that a group of chemokines shows higher elevations at 72 h in the circulation of patients destined to remain critically ill.

### Specific immune mediator and receptor families mirror overall immune mediator patterns

**Figure 5:**
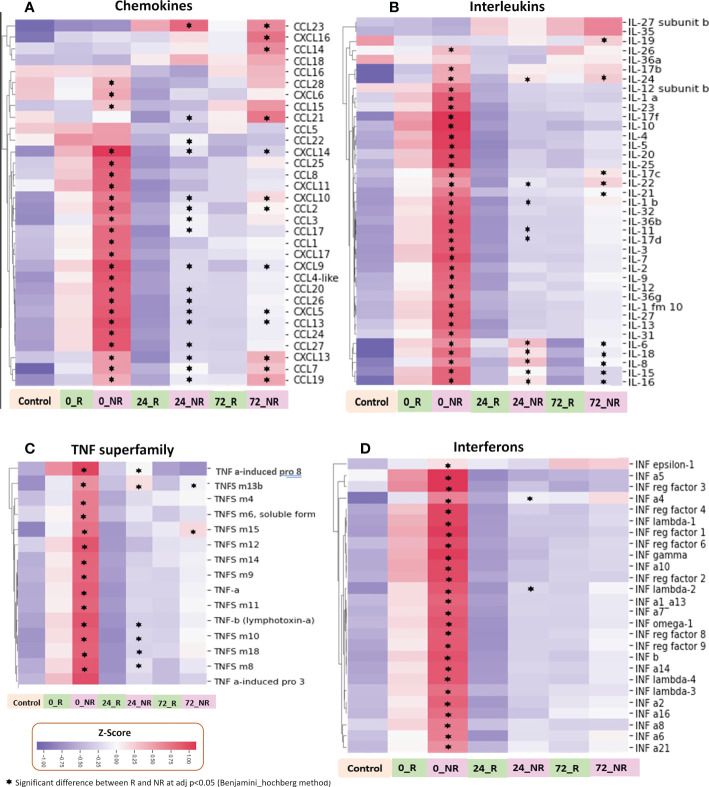
Heatmaps illustrating normalized z-scores for chemokines **(A)**, interleukins **(B)**, TNF superfamily **(C)**, and interferons **(D)** in ISS matched NR (n=39) and R (n=26) patients at 0, 24 and 72 h with low ISS (ISS of 1 or less, n=29) patients as controls.

To provide further detail on the mediator families included in this analysis, we show the heatmaps for each of the four mediator families, including chemokines, cytokines, interferons, and TNFSF members, comparing ISS, age, and sex matched NR and R patients (**Figure 5**). Mediators marked with an asterisk are significantly different, with an adjusted *p*-value <0.05, when comparing NR to R patients. The SomaLogic assay includes 33 of the 47 known chemokines (**Figure 5A**). At 0 h in both groups, 18 of the 33 chemokines were higher in comparison to the low-ISS control patients, and these levels were obviously higher in NR patients in comparison to R patients. There was a subset of chemokines that were low in both groups at 0 h but were elevated at 72 h and to a significantly greater extent in the NR group including CCL14, CCL23, and CXCL16. Other chemokines significantly higher in NR vs. R at 72 h were CCL 2, 7, 13, 19, and 21 and CXCL 5, 9, 10, 13, and 14.

All 21 known interferons, 15 of 19 known human TNF superfamily members, and 31 of 40 known human interleukins (multiple members or subunits for some interleukins) were identified in the SomaLogic panel. Interleukin, interferon, and TNF superfamily mediators showed an early broad release at time 0 h in NR and R groups, however, the patterns at 24 and 72 h were less dramatic than those within chemokines (**Figures 5B-D**). There were notable differences between NR and R patients within the interleukin family, with a small subset of mediators high only in NR patients in comparison to R patients at 24 and 72 h, including, IL-6, IL-15, IL-16, IL-19, and IL-24 (**Figure 5B**). Within TNF superfamily mediators, there was broad massive release at 0h in the NR patients with almost complete dropout at 24 and 72 h. Proteins in this mediator family did not demonstrate notable delayed selective increases in NR patients (**Figure 5C**).

In addition to mediators, receptor families also demonstrated interesting patterns in the circulation following trauma. Heat maps for soluble interleukin, TNF, and interferon receptors are shown in **Supplemental Figure 2**. Receptors marked with an asterisk were significantly different, with an adjusted *p*-value <0.05 when comparing NR to R patients. The SomaLogic panel includes 18 of the 21 known interleukin receptors (comprised of 37 subunits), 20 of the 27 known TNF superfamily receptors, and all 4 interferon receptor subunits. Most of these were increased in circulation at 0 h in both groups in comparison to the low ISS controls, but to a greater extent in NR patients. Heatmaps demonstrating normalized z-scores for interleukin and TNF superfamily receptors are shown in **Supplemental Figures 2A, B**, respectively. Selective increases at 24 and 72 h are notable for IL-1 and IL-3 receptor subunits, and TNFRS m1, m10, and m11 subunits. These findings suggest that there is broad release of surface receptors early after severe injury with more selective shedding at later time points in patients with a prolonged recovery.

### Mediator levels at 72h accurately predict patient outcomes

**Figure 6:**
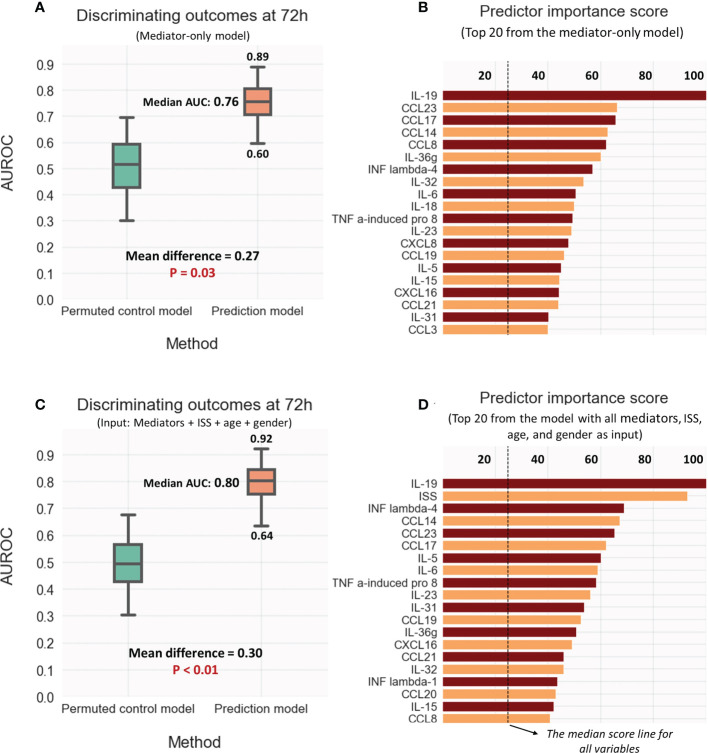
Discriminative model performance and strongest predictors of outcome (Resolver vs. Non-resolver) at 72hr: **(A, B)** Using mediator values only; **(C, D)** Using the values for ISS, Age, and sex as well as the mediators. Discriminative performance compared to the control model was measured by calculating the area under ROC curve (AUROC); the strongest predictors were selected as rated by generic predictor importance score (see methods).

As shown in **Figure 6A**, the LASSO-SVM prediction model that utilized 72h mediator values predicted patient oucomes significantly better than an imputed control model (median AUROC 0.76, p-value 0.03). IL-19 and the chemokines CCL23, CCL17, CCL14 possessed the highest prediction scores and were placed higher than IL-6 (**Figure 6B**).

While the addition of ISS, age, and gender minimaly improved the predictions (median AUROC 0.8, P-value < 0.01), the overall model performance and predictor scores were mostly unchanged (**Figures 6C, D**). Interestingly, IL-19 remained the strongest feature with a predictor score higher than that of ISS. **Supplemental Figures 4A, B** demonstrating visualizations of PLS-DA for the predictive models reinforce the notion that almost of all of predictive information in ISS is adequately captured by the set of mediator values.

### Mediator patterns can be quantitatively validated in an external dataset

**Figure 7:**
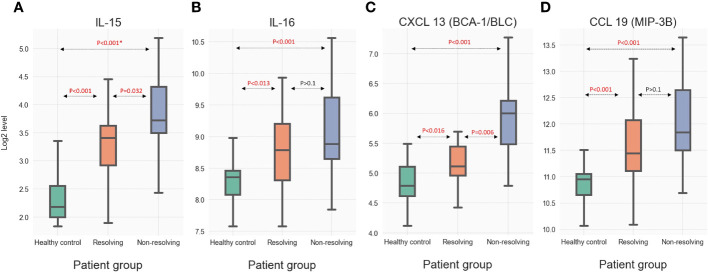
Quantitative measure of selected immune mediators including **(A)** IL-15 **(B)** IL-16 **(C)** CXCL 13 and **(D)** CCL29 in circulation of VC at 72 h as analyzed by the MSD assay. * For each mediator, the presented p-values were calculated through Dunn's post-hoc method. Then, the Benjamini-Hochberg method was used to further adjust the p-values from multiple tests.

To validate the novel observations made through the analysis of the SomaLogic proteomic database, we carried out additional quantitative analyses for four immune mediators utilizing an external trauma database and biobank. This validation cohort (VC) was established by selecting a subset of patients (n=58) from a previously published trauma database (13, 31) after matching the clinical characteristics of the R and NR groups from the experimental PAMPer patients. Samples from these VC patients along with the samples from the experimental PAMPer cohort and healthy controls (n=20) underwent quantitative analysis using the MSD platform. The patient demographics and outcomes of the VC (comprised of 33 R and 25 NR patients) and experimental PAMPer cohorts are shown in **Supplemental Table 4**. Samples from the PAMPer patients used for the SomaLogic analysis as well as samples from the VC patients have had targeted quantitative immune-mediator analysis performed previously using the Luminex™ platform (8). To assure that these cohorts were indeed similar in their quantitative mediator response patterns, we compared the Luminex™ data for IL-6 and IL-8 (CXCL8), mediators identified as significantly elevated in our SomoaLogic data and known to be elevated after injury (7). As shown in **Supplemental Figure 3**, levels at 0, 24, and 72 h were elevated to a similar degree between the PAMPer and VC R, and NR patient subsets. Thus, VC patients are a representative population for quantitative validation of the PAMPer cohort findings. **Figure 7** includes boxplots demonstrating quantitative MSD data for four (two cytokines and two chemokines) immune mediators in the circulation of VC patients that were found to be significantly elevated in the SomaLogic analysis of the PAMPer patients at 24 and 72 h. Significant increases were observed in the R and NR subsets of the matched VC patients at 72 h for IL-15 (6A), IL-16 (6B), CXCL13 (6C), and CCL19 (6D) above the healthy controls. Further significant increases in NR vs. R patients were observed for IL-15 and CXCL13. These two cross-platform data analyses of two unique datasets provide a strategy to validate mediator patterns.

## Discussion

In response to injury, there is an immediate activation of immune responses that are implicated in the development of a complicated post-trauma clinical course. In this study, we extend our understanding of the immune response to injury by analyzing the circulating patterns of 115 protein immune mediators and 69 soluble receptors in severely injured humans in a temporal manner. This analysis yielded several novel findings. First, there is a broad release of both immune mediators and mediator receptors early after injury. This release is greater in patients that die early or recover slowly and associates with injury severity. Second, the mediator patterns evolve rapidly over the first 72 h with selective elevations of several mediators known to correlate with outcomes (i.e., IL-6 and CXCL8) as well as mediators not previously studied in human injury (e.g., CXCL13, IL-15). Elevations in these mediators are notably greater in patients that recover slowly. Our findings provide the most comprehensive view of the circulating immune mediator landscape early after injury reported to date and implicate biological processes such as the passive release of mediators and receptors early followed by chemokine-driven leukocyte trafficking at 24-72 h. Furthermore, our findings provide insight into the temporal development of the immune response to trauma by evaluating key mediators and receptors from admission through 72 h.

One limitation to the measurement of circulating immune mediators in any disease process is the availability of assays. We and others have used single-mediator assays such as ELISA as well as multiplexing assays to measure temporal changes in up to 32 immune and inflammatory mediators simultaneously (8–10, 13, 16, 33–36). Current paradigms are based on descriptions of the mediators that have been measured in these assays. For example, it is well-established that IL-6, IL-8, IL-10, and MCP-1 (CCL2) are elevated early after injury and are correlated with adverse outcomes including nosocomial infection, multiple organ dysfunction syndrome, and mortality (8, 31, 36). In addition, delayed adverse outcomes are associated with a cytokine signature associated with a pathogenic Th17 response (IL-17A + GM-CSF) (8, 10, 17). In studies using multiplexing assays of up to 16 mediators, we have noted that cytokines and chemokines elevated after injury can be separated into three groups based on similar patterns of elevation. These groups can be categorized as pro-inflammatory, lymphocyte-associated, and protective/reparative. It has been shown that in the early response to severe injury, the pro-inflammatory group of cytokines is increased while the reparative/protective group of cytokines is suppressed (16). However, patients that recover early have an increase in the reparative and protective mediators on admission (17). The SomaLogic assay measures levels of all known interferons (n=21), 31 of 40 known interleukins (including multiple members or subunits for some interleukins), 33 of the 47 known chemokines, and 15 of the 19 known TNF superfamily mediators. The SomaLogic assay has been validated in large cohorts of human subjects and has been widely used as the proteomic platform to establish circulating proteomic patterns in disease (25, 37, 38). Important to note is the sensitivity of the SomaLogic assay to changes in protein concentration. The significant fold change difference in several mediators represents a narrow change in concentration for many mediators. In this setting, the utility of this proteomic data is in identifying novel patterns of mediator increase or decrease, and using this information to inform further validation studies, which may be quantitative in nature. Our strategy of screening for novel mediator patterns using a large scale proteomics platform followed by validation using a second analysis platform could be adopted to fully analyze circulating regulatory molecules in the human injury response.

Perhaps the most striking observation of the present work is that within this broad characterization of the immune response, nearly all the assessed mediators and surface receptors are elevated in the circulation of injured patients in comparison to controls at 0 h. We demonstrate that the degree of elevation is dependent both on patient outcome and injury severity, with non-resolving patients and those severely injured having greater elevations in mediator and receptor levels. This suggests a massive release of mediators and cell surface receptors very early in response to traumatic injury. While it remains unclear if this release is active or more passive in nature, it may represent a maladaptive response with resultant cellular confusion and immune dysfunction that is more prominent with increasing injury severity and in those with a prolonged clinical course.

Within the broad cohort of immune mediators that are elevated early in the response to injury, a small subset of these remains elevated at 24 and 72 h. Furthermore, a subset that is low at time 0 h is elevated at 24 and 72 h. This suggests a temporal shift of the response as the patient progresses from 0h to 72h, and potentially a reprogrammed inflammatory state. This delayed selective increase of mediators is associated with outcome, with non-resolving patients demonstrating greater increases than resolving patients. However, this phenomenon is not dependent on injury severity, with similar delayed increases in patients with mild, moderate, or severe ISS. Thus, this subset of mediators may represent those that promote ongoing immune dysfunction in non-resolving patients. We additionally have performed a limited number of quantitative assays to validate these findings in an external dataset. This allowed us to validate the pattern of delayed selective increase of immune mediators in the circulation of non-resolving patients.

When critically examining this subset of mediators with delayed elevations in non-resolving patients, we find that cytokines and chemokines are over-represented in this subset. Furthermore, many of these cytokines and chemokines (or chemotactic cytokines) have been largely unstudied in the field of trauma. These mediators play an important role in immune cell trafficking to secondary lymphoid organs such as lymph nodes and thymus, and also are involved in priming and activation of cells within the innate and adaptive immune systems (7, 39–41). Thus, a sustained increase of these mediators at delayed timepoints in non-resolving patients may promote the ongoing immune activation and prolonged inflammation that contributes to a complicated clinical course.

TNFSF members demonstrate a unique pattern in comparison to what is described for the three mediator families in this analysis. TNFSF members demonstrate early massive release in the NR patients to a much greater degree than R patients, however not seen is a delayed selective increase at 24 or 72h. Lack of increase of this generally pro-inflammatory mediator at delayed timepoints argues for the potential development of disease resilience in both R and NR patients.

There are several limitations of this work. The observations are largely correlative in nature and will require further validation in a prospective manner to utilize these findings in a prognostic manner. In particular, the predictive capabilities of specific mediators do not necessarily indicate a causal or biologically significant role in the post-trauma immune response. In any case the fact that the strength of IL-19 contributions to outcome prediction in the PAMPer population was more substantial than ISS and IL-6 highlights this mediator as a potential area of focus for future investigations.

Additionally, the mechanisms of mediator and receptor elevation in the circulation could not be established through this work and require further study to determine if they are active or passive in nature. Also, the inclusion of the selected immune mediator families is not a complete evaluation of all immunomodulatory proteins. Further work using these methods to analyze proteins such as damage associated molecular pattern molecules (DAMPs), complement mediators, and antibodies would add value to this work. Finally, the functional impact of the observed circulating immune mediators and receptors requires further investigation.

In conclusion, simultaneous analysis of a large number of circulating immune mediators and soluble receptors in trauma patients demonstrates previously unappreciated patterns in the elevations of these proteins. Many more mediators and their soluble receptors are increased early after injury than known before. This broad elevation in mediators gives way to increases in a subset of several cytokines and chemokines, most not previously measured in the setting of trauma. The association of these patterns with adverse outcomes suggests that many of the mediators may contribute to the immune dysfunction known to occur after severe injury and should be investigated further.

## Data availability statement

The original contributions presented in the study are included in the article/**Supplementary Material**. Further inquiries can be directed to the corresponding author.

## Author contributions

IB, JW, UK, and TB designed the workflow and defined analysis strategies. IB, JW, HM, UK, SL, JB, and RN performed data analysis and visualization. IB, JB, HM, and TB wrote the manuscript with feedback from all the listed authors who have read and approved the manuscript. JS, MN, TB designed the original study and sampling plan. PAMPer and STAAMP study authors contributed to patient enrollment and sample procurement. All authors contributed to the article and approved the submitted version.

## Funding

This work was supported by the US Army Medical Research and Material Command (W81XWG-12-2-0023 to JS) and the National Institutes of Health (R35-GM-127027 and T32-GM-008516 to TB, R01LM012087, and R35-GM-119526 to MN, R38-HL-150207 supporting JB). The University of Pittsburgh holds a Physician-Scientist Institutional Award from the BurroughsWellcomeFund-JB. JW was a visting scholar supported by Central South University, Changsha, China.

## Acknowledgments

We acknowledge the contributions of collaborators involved in the PAMPer and STAAMP trials for their efforts in patient enrollment, sample procurement, and clinical data collection.

## Conflict of interest

YV is co-founder of, and stakeholder in, Immunetrics, Inc.

The remaining authors declare that the research was conducted in the absence of any commercial or financial relationships that could be construed as a potential conflict of interest.

## Publisher’s note

All claims expressed in this article are solely those of the authors and do not necessarily represent those of their affiliated organizations, or those of the publisher, the editors and the reviewers. Any product that may be evaluated in this article, or claim that may be made by its manufacturer, is not guaranteed or endorsed by the publisher.
