# Supplementary figures and images for: Plasma proteomics reveals early, broad release of chemokine, cytokine, TNF, and interferon mediators following trauma with delayed increases in a subset of chemokines and cytokines in patients that remain critically ill
Source: Front Immunol. 2022 Nov 30;13:1038086. doi: 10.3389/fimmu.2022.1038086 (PMC9750757; doi:10.3389/fimmu.2022.1038086)

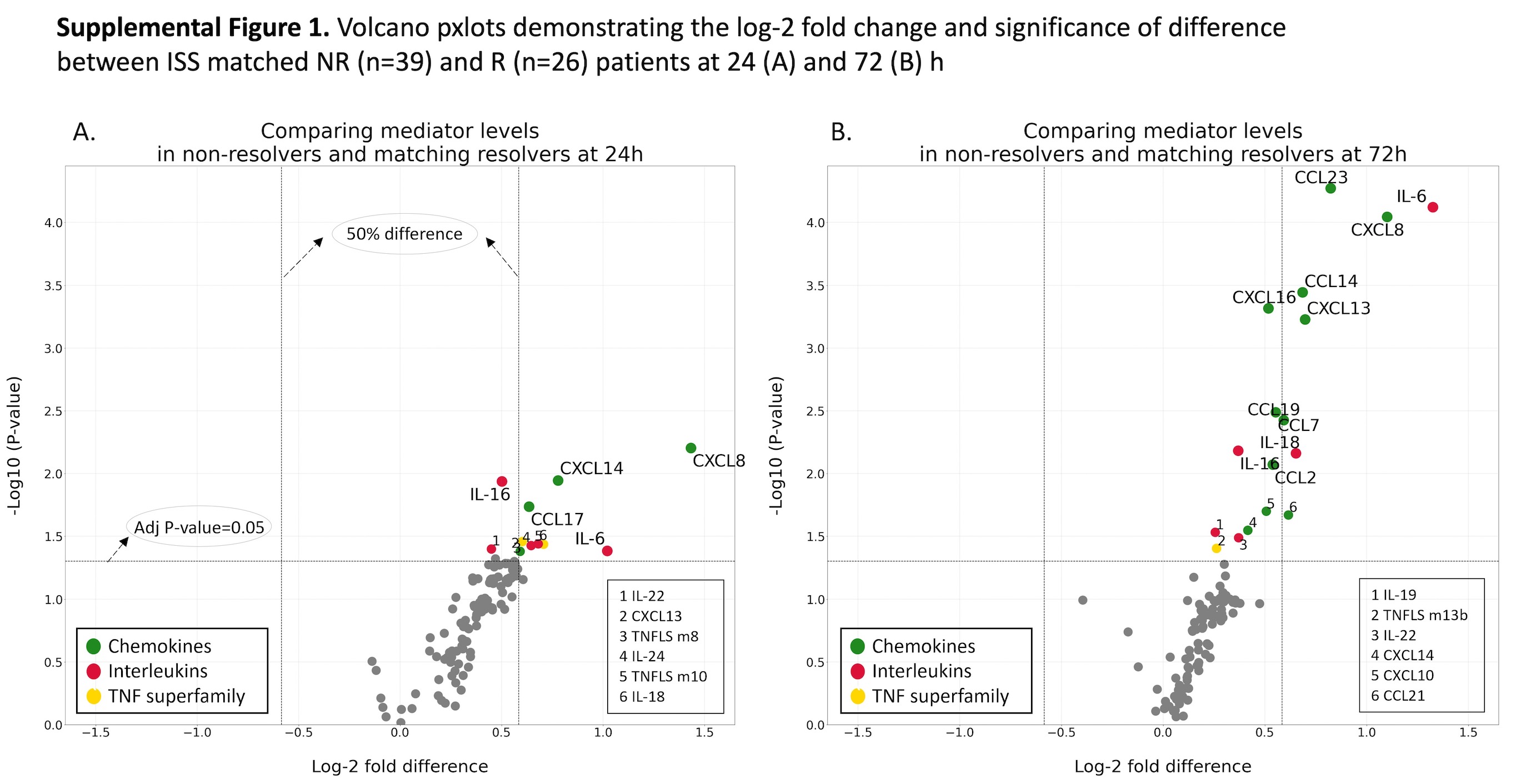

Supplement: Supplementary file 1 [file Image_1.jpeg]

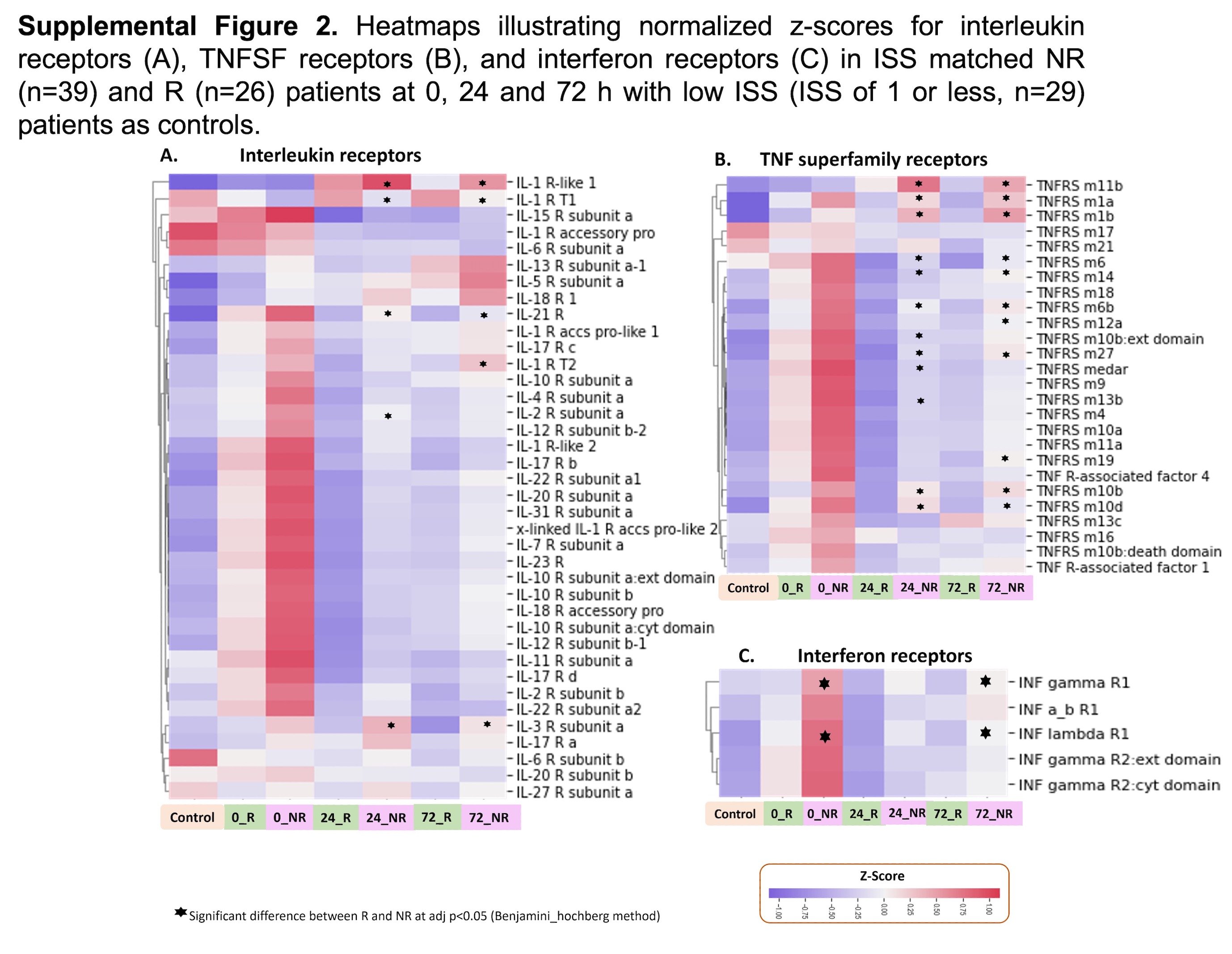

Supplement: Supplementary file 2 [file Image_2.jpeg]

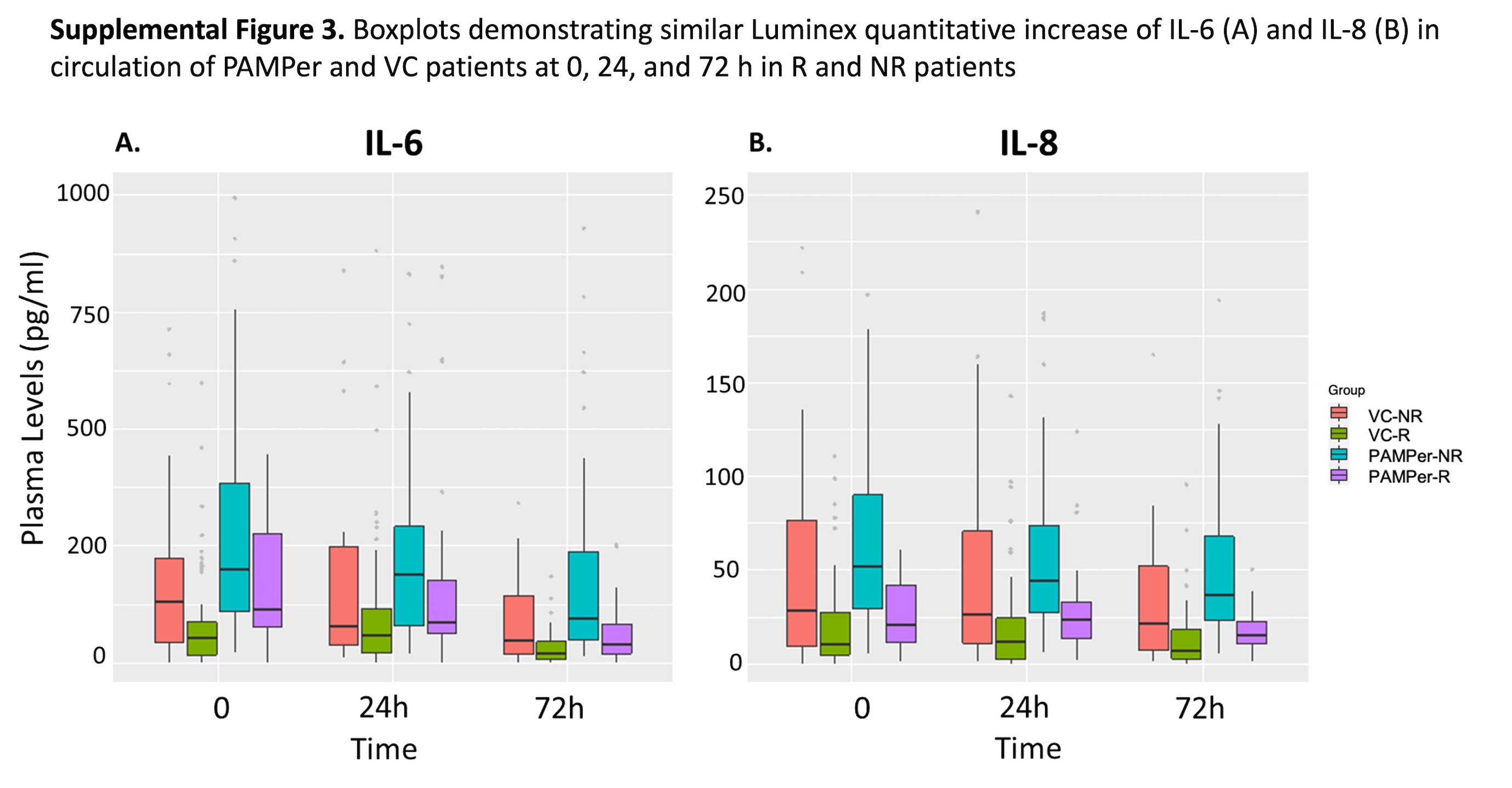

Supplement: Supplementary file 3 [file Image_3.jpeg]

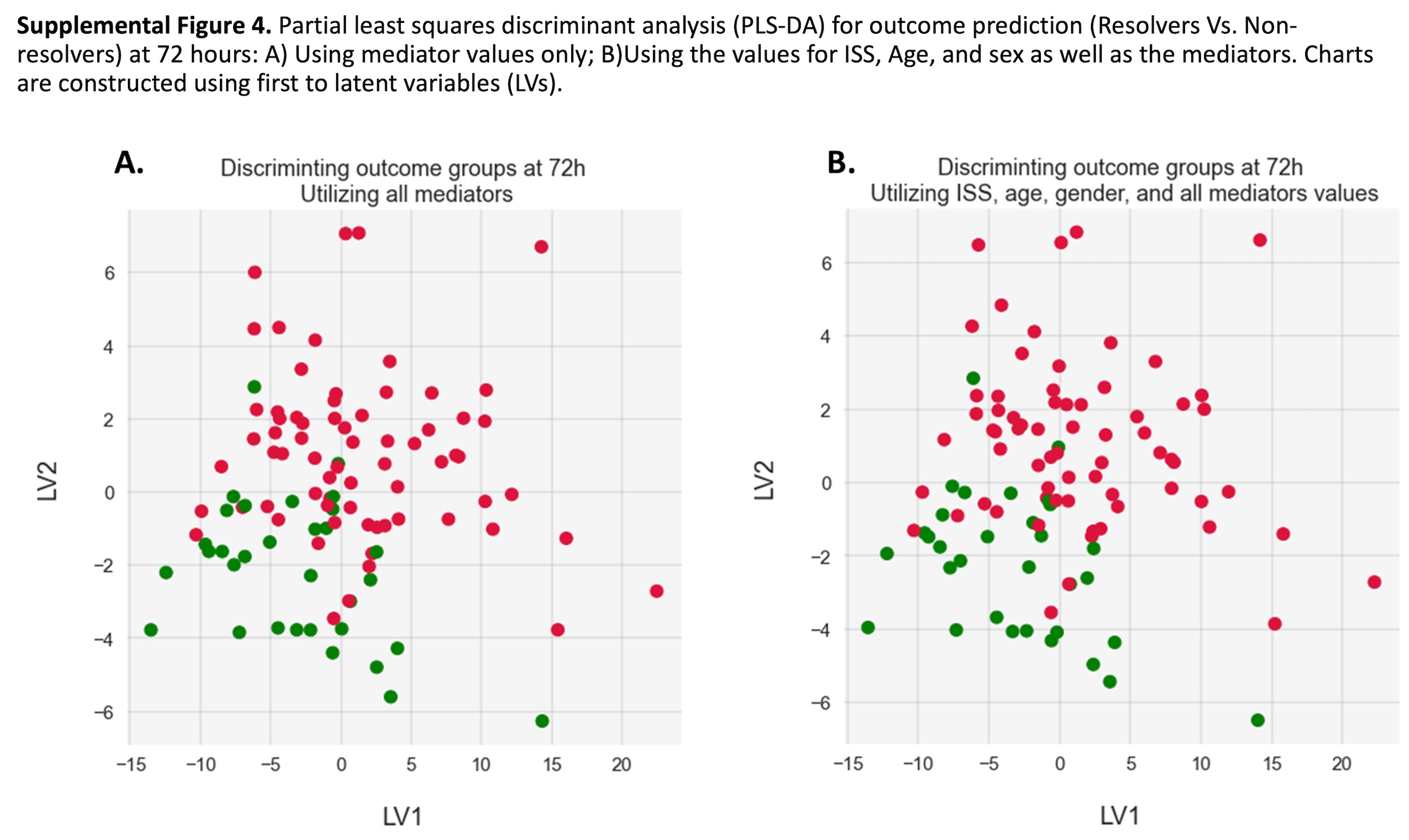

Supplement: Supplementary file 4 [file Image_4.jpeg]

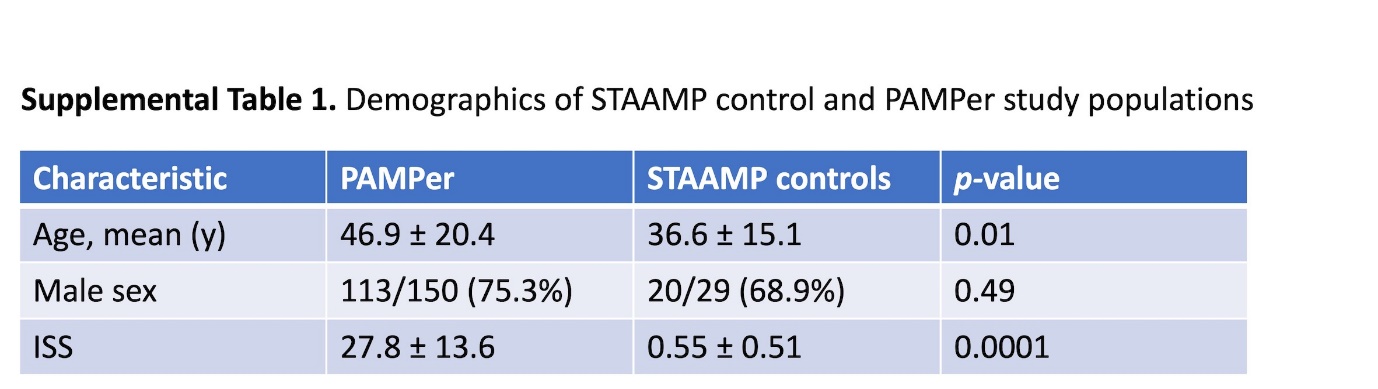

Supplement: Supplementary file 5 [file Table_1.docx]

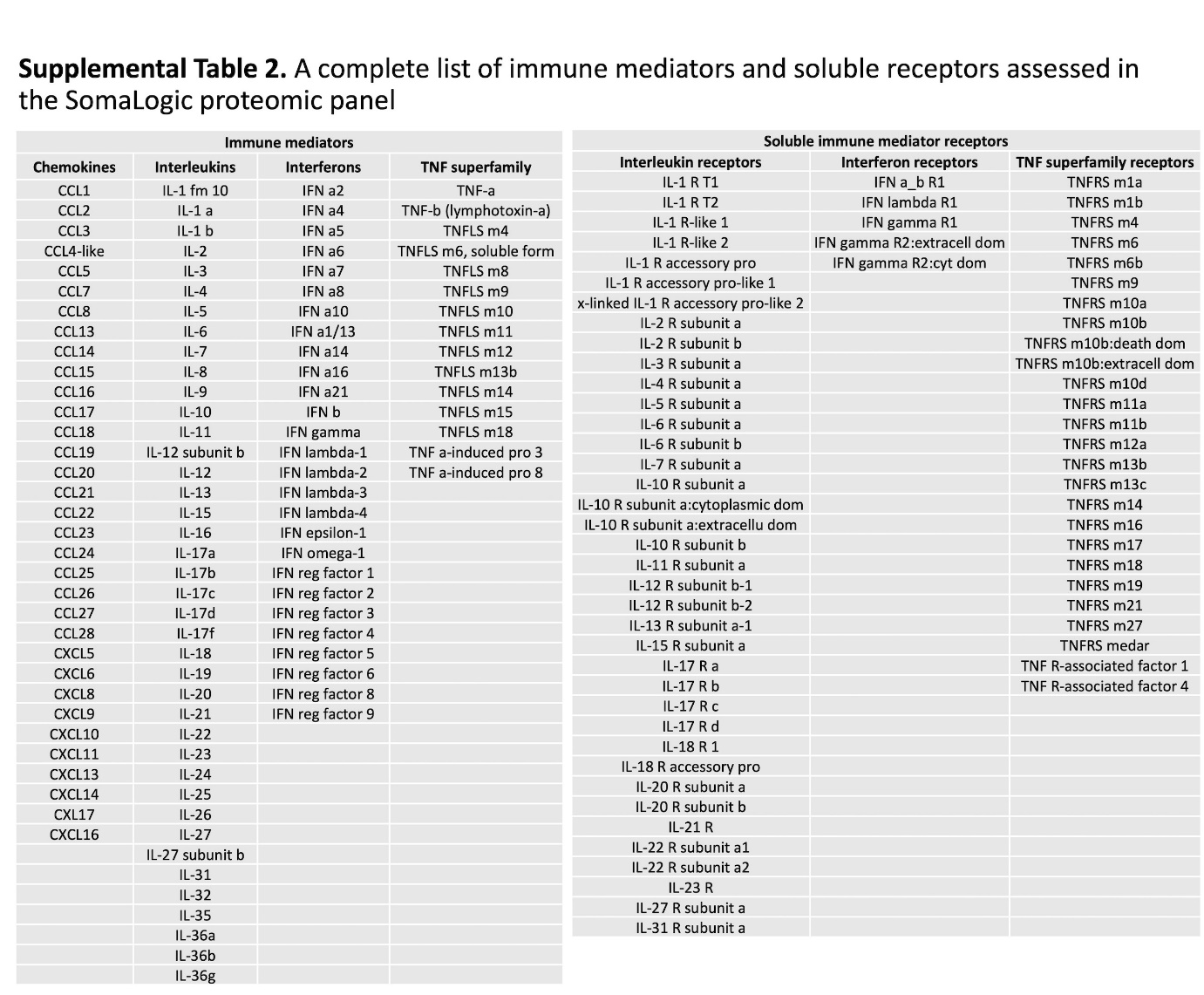

Supplement: Supplementary file 6 [file Table_2.docx]

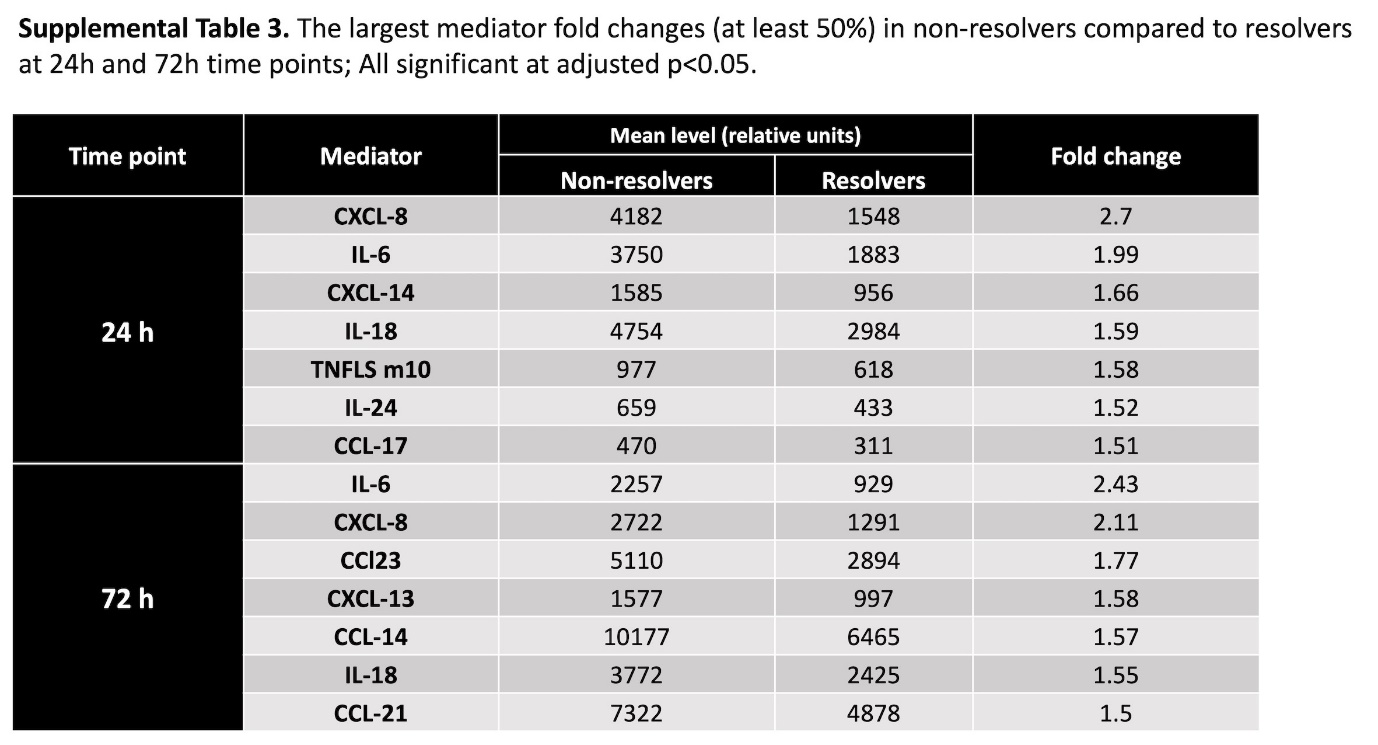

Supplement: Supplementary file 7 [file Table_3.docx]

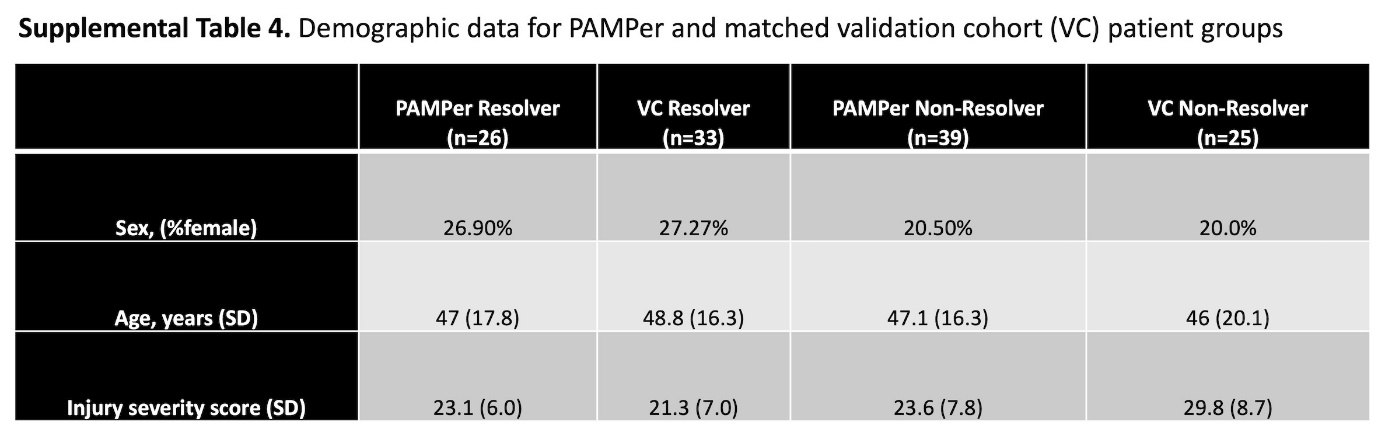

Supplement: Supplementary file 8 [file Table_4.docx]
